# A Federal Indian Health Insurance Plan: Fulfilling a solemn obligation to American Indians and Alaska Natives in the United States

**DOI:** 10.1016/j.pmedr.2021.101669

**Published:** 2021-12-16

**Authors:** Vishal Khetpal, James Roosevelt, Eli Y. Adashi

**Affiliations:** a Department of Internal Medicine, The Warren Alpert Medical School, Brown University, Providence, RI, USA; b Department of Public Health and Community Medicine, Tufts University School of Medicine, Boston, MA, USA; c The Warren Alpert Medical School, Brown University, Providence, RI, USA

**Keywords:** American Indian health, Indian Health Service, Public health insurance, Health disparities

## Abstract

The *Indian Health Service* (IHS) is but the latest effort to honor the constitutional and treaty commitment made to deliver health care to American Indians and Alaska Natives in the United States. The agency currently serves over 2.5 million American Indians and Alaska Natives through IHS-operated health facilities, as well provides funding support for contractual delivery of health services by Tribes to their constituents. Not unlike its predecessors, however, the IHS is struggling to fulfill its stated mission. Access to specialty care remains limited through the agency, particularly for Urban Indians, due to limited fiscal support of the Purchase-Referred Care Program. As a result of chronic underfunding stemming from Congressional inaction, the agency faces perennial understaffing and leadership turnover. In addition, the IHS is structurally unprepared to address the progressive urbanization of Alaska Natives and American Indians, more of whom now live in New York City than in North Dakota. Given these challenges, a renewed approach is warranted. In this essay, we review the history of the IHS, examine its current fiscal challenges, outline the ongoing demographic shift of AI/AN toward urban centers, and call for the creation of a Federal Indian Health Insurance Plan. Offered as a benefit to all American Indians and Alaska Natives without cost-sharing, the plan would be applicable both on and off tribal lands. Funded through mandatory appropriations, the plan would significantly increase fiscal support for AI/AN health programs and work to reduce health disparities affecting AI/ANs in the United States.

## Highlights

• The U.S. has a legal obligation to deliver health care to American Indians and Alaska Natives (AI/AN).
• The Indian Health Service is the federal agency tasked with fulfilling this mission.
• The American Indian and Alaska Native population is rapidly urbanizing.
• The agency is chronically underfunded and faces new, significant demographic shifts.
• We propose a Federal Indian Health Insurance Plan, extending coverage to all AI/AN.

The *Indian Health Service* (IHS) is the federal agency tasked with raising the “physical, mental, social, and spiritual health” of American Indians and Alaska Natives (AI/ANs) in the United States. Not unlike its predecessors, the IHS is struggling to fulfill its stated mission. The *U.S. Government Accountability Office* has found the IHS to be in a never-ending state of crisis, marked by budgetary shortfalls, perennial understaffing, and frequent leadership turnover (Indian Health Service: Spending Levels and Characteristics of IHS and Three Other Federal Health Care Programs (No. GAO-19-74R), 2018, Levinson, 2016). Reports of sewage leaks in hospital operating rooms and of poorly stocked emergency resuscitations carts further affirmed the dire state of Indian health care (Levinson, 2016). Persisting health disparities, left unaddressed and exacerbated by the COVID-19 pandemic, have contributed to higher rates of type 2 diabetes mellitus, kidney disease, and cirrhosis than national averages. Overall, AI/AN have a life expectancy that is over 5 years less than the general population (Burki, 2021, “IHS Profile | Fact Sheets,” 2021). Apart from these considerations, the IHS is hard pressed to address the progressive urbanization of American Indians and Alaska Natives, more of whom now live in New York City than in the rural state of North Dakota, home to a historically large AI/AN community (Norris et al., 2010). A renewed approach is necessary to fulfill the constitutional and treaty commitment made to the American Indians and Alaska Natives today. In this essay, we review the history of the IHS, examine its current challenges, and call for the creation of a Federal Indian Health Insurance Plan, to work alongside the IHS, that is applicable both on and off the tribal lands.

The IHS can trace its origins to the 17th century outbreaks of smallpox and measles, which all but decimated American Indian communities (Bergman et al., 1999, Warne and Frizzell, 2014). Overtaken by disease, the Indian tribes of the day took to bartering resources in return for whatever limited health services could be afforded by the early colonists (Warne and Frizzell, 2014). It was not until 1787 that the U.S pledged health care to American Indians by way of the *Indian Commerce Clause* of the Constitution (Bergman et al., 1999, Warne and Frizzell, 2014). Fortified by multiple subsequent treaties, case law, statutory law, and executive orders, the *Indian Commerce Clause* remains the legal bedrock undergirding the mission of the IHS and its predecessors (Levinson, 2016, Warne and Frizzell, 2014). The first federal agency designated to uphold the “federal Indian trust responsibility” for health care, and the predecessor of the modern Indian Health Service, was the *Bureau of Indian Affairs* (BIA) (Trout et al., 2018, Warne and Frizzell, 2014). Limited by design to disease containment, the BIA, a constituent of the Department of the Interior, proved incapable of providing general individual health care. It was not until the enactment of the *Indian Citizenship Act of 1924* that BIA appropriations were specifically targeted “for relief of distress and conservation of health” (Bergman et al., 1999, Trout et al., 2018). It took the *Indian Health Facilities Act (Transfer Act) of 1954* to see to the transfer of the Indian health program to the Public Health Service (Department of Health, Education, and Welfare) and thereby to the establishment of the modern IHS (Bergman et al., 1999, “IHS Profile | Fact Sheets,” 2021, Trout et al., 2018, Warne and Frizzell, 2014).

Today’s IHS is charged with the provision of health care services to over 2.5 million American Indians and Alaska Natives. In the 2010 United States Census, 5.2 million Americans self-identified as AI/AN, alone or in combination with another race. Eligibility is determined by membership to one of 574 federally-recognized Tribes, though other limited criteria exist for eligible non-Indians (“IHS Profile | Fact Sheets,” 2021). The subject of discretionary congressional appropriation ($6.0 Billion in FY20), the IHS, unlike Medicaid or Medicare, does not constitute an earned benefit, nor is it the beneficiary of mandatory spending (Indian Health Service (IHS) fiscal year (FY) 2020 congressional justification, 2019). Subject to these limitations, the IHS is entrusted with the employment of health care personnel and with the operation of multiple inpatient and outpatient facilities nationwide (“IHS Profile | Fact Sheets,” 2021, Warne and Frizzell, 2014). The agency also administers public health programs such as the *Special Diabetes Program* with an eye toward addressing established health care disparities (“IHS Profile | Fact Sheets,” 2021, Indian Health Service (IHS) fiscal year (FY), 2020). Through the *Indian Self-Determination and Education Assistance Act,* passed in 1975, Tribes have been allowed to administer and deliver health services through contractual funding agreements with the federal government (Bergman et al., 1999, “IHS Profile | Fact Sheets,” 2021, Indian Health Service (IHS) fiscal year (FY), 2020, Warne and Frizzell, 2014). Within this framework, a select number of Tribes have been able to supplement federal contract funding with gaming revenue (Wolfe et al., 2012). Today, over 60% of the appropriated IHS budget is contracted to and administered by the Indian Tribes (“IHS Profile | Fact Sheets,” 2021, Indian Health Service (IHS) fiscal year (FY), 2020).

The IHS faces a challenge of chronic underfunding, as the IHS budget in recent years has not been adequate to meet the needs of the program. Congressional inaction, alongside fiscal scarcity, compounded by growing beneficiary rolls, precludes the IHS from living up to its obligations. Partial budgetary relief ($1.2 Billion in FY19) is afforded by the agency’s status as a *payer of last resort*. This status compels that care-related costs are to be assumed by a patient’s other eligible health insurance funding sources, such as private insurance, Medicare, Medicaid, the Children’s Health Insurance Program (CHIP), and the Department of Veterans Affairs before the IHS itself covers these costs from funds directly allocated to the agency (Indian Health Service (IHS) fiscal year (FY), 2020, Indian Health Service: Spending Levels and Characteristics of IHS and Three Other Federal Health Care Programs (No. GAO-19-74R), 2018). The agency has also received significant financial relief from the Coronavirus Aid, Relief, and Economic Security Act and the American Rescue Plan Act of 2021 during the COVID-19 pandemic. Yet the per-capita health care spending levels of the IHS in 2019 ($4,078) still paled in comparison to Medicaid ($8,109), the Veterans Health Administration ($10,692), and Medicare ($13,185), underscoring the agency’s funding challenges rooted in insufficient budget allocation by Congress. (Indian Health Service: Spending Levels and Characteristics of IHS and Three Other Federal Health Care Programs, 2018). Underfunding has contributed to longstanding issues facing the IHS in personnel recruitment, quality oversight of hospitals by the organization, and in implementation of updated information technology resources (Levinson, 2016). Contending with limited resources, the IHS is often forced to withhold key health care services. The Purchase-Referred Care Program (PRCP) is a case in point (Levinson, 2016). Designed to compensate IHS enrollees for out-of-network services, the perpetually underfunded PRCP is frequently unable to live up to its mandate except in extreme circumstances such as the imminent loss of life (Levinson, 2016). Furthermore, the program is often utilized to access common diagnostic tests in specialty care, beyond the scope of the primary care focus of most IHS facilities. It is this all-too-familiar budgetary exhaustion, well before the end of the fiscal year, that underlies the oft-quoted Indian country phrase “don’t get sick after June.”

Apart and distinct from its budgetary challenges, the IHS is contending with a significant demographic shift in the AI/AN population. In 1954, at the inception of the IHS, at least 70% of AI/ANs lived on the tribal lands (Norris et al., 2010, Snipp et al., 1996). Today, however, 70% of AI/ANs live in urban settings wherein many are still subject to the same leading causes of death as their rural counterparts (Burki, 2021, Norris et al., 2010). Cognizant of this trend and its likely accentuation in time, the Office of Urban Indian Health Programs of the IHS contracted with 41 Urban Indian Organizations in an effort to provide health services for Urban Indians throughout the United States (Indian Health Service (IHS) fiscal year (FY) 2020 congressional justification, 2019). These entities operate alongside traditional IHS service units, which facilitate delivery of health services within specific geographic areas through one or more facilities. Service units are operated both by the IHS and by Tribes themselves, with a particular focus often in primary care. In contrast, Urban Indian Organizations (UIOs) are administered through a separate office, and may offer a significant, unpredictable variety in health services. Although most provide direct medical care, some alternatively focus on behavioral health counseling and general health education (The Indian Health Service (IHS): An Overview, 2016). Furthermore, much of the annual budget of the IHS remains targeted at health care services based in tribal lands (Indian Health Service (IHS) fiscal year (FY) 2020 congressional justification, 2019). Funding emphasis toward services based in tribal lands is justifiable, due to higher costs related to providing health services in this setting. It is also historically appropriate, as the federal government’s trust responsibility was forged with the Tribes, rather than individuals. Fully funding the current IHS through mandatory appropriations, as well as undertaking substantial organizational reform, would certainly help the organization better fulfill it stated mission. However, the ongoing demographic shift warrants the pursuit of alternative models of payment and health care delivery for AI/AN no longer residing on traditional tribal lands.

In recent years, the growing recognition for a shifting status quo in Indian health has spawned a search for alternative models to better fund access to health services for AI/ANs. One example includes the prospect of a Navajo Nation Medicaid agency (Report to congress on the feasibility of a Navajo nation Medicaid agency, 2014). Another proposal called for the replacement of the IHS with a CHIP-like block grant model, empowering Tribes to fashion their own health care system (Frias, 2003). Neither model, however, comports with the legal commitment made to American Indians and Alaska Natives, given the likelihood of cost-sharing between the Tribes and the federal government.

We therefore propose a benefit program in the form of a Federal Indian Health Insurance Plan offered to all AI/AN as a right, to work alongside the IHS. Covering the entire cost of health insurance to its beneficiaries, the proposed plan would address the demographic challenge by making it possible for eligible AI/ANs to affordably seek care outside of IHS facilities, regardless of geography. In doing so, the program would facilitate more robust access to specialty and emergency care services. This would particularly be the case for Urban Indians in proximity to alternative options for seeking health services who may currently utilize the PRCP for these services. By focusing on health care delivery through an alternative payment model for AI/ANs, the Federal Indian Health Insurance Plan would offer significant financial support for the current IHS, while allowing for the organization to focus its efforts on facilitating high-quality health care delivery to AI/ANs primarily residing on tribal lands through its current hospitals, clinics, and health stations.

The Federal Indian Health Insurance Plan would build upon the progress made by the Affordable Care Act (ACA). The ACA offered significant Indian-specific provisions, such as greater flexibility in health insurance enrollment in the individual marketplace exchanges, limited or elimination of cost-sharing for health plans based on income, improved reimbursement to IHS hospitals through Medicare, and promotion of traditional healing practices. The legislation additionally facilitated the expansion of Medicaid, to the benefit of many AI/AN individuals. We envision that the Federal Health Insurance Plan could further expand on these benefits for AI/ANs. The program would be offered to AI/ANs without premiums or other forms of cost-sharing regardless of income, in line with the historic and legal commitment of the federal government to deliver health services as a benefit. To beneficiaries, it would offer a targeted, culturally competent health insurance product. We envision that the program would offer significant, directed reimbursement for traditional healing practices, which are only offered limited coverage, up to $300 per year, under few Medicaid plans currently (Medicaid’s Role in Health Care for American Indians and Alaska Natives, 2021). Tribal working groups and other key stakeholders can specifically mold the benefits package for the program. To ensure accessibility to sites of health care delivery such as clinics and hospitals, the federal government can mandate providers accepting Medicare or Medicaid to also participate in this program.

Funding for the program, provided through mandatory Congressional appropriation, would increase in proportion to the population growth of its beneficiary pool over time to match the average annual per-capita spending of Medicaid (currently $8,109). Mandatory funding would also ensure lasting political stability in the fiscal health of the insurance program. As many do today, Tribes could continue to deliver health services to their constituents, bolstered by an insurance program supported by a stable source of funding from Congress alongside other revenue streams. Similarly to the Tribes, the IHS would also continue to deliver health services through its existing facilities, with a particular focus for AI/ANs residing on tribal lands. If implemented today, the program would receive funding to $19 billion. Combined with current IHS appropriations, total funding for AI/AN communities would increase to $26 billion – a figure that is over four times greater than appropriations before the pandemic (Indian Health Service (IHS) fiscal year (FY), 2020, Indian Health Service: Spending Levels and Characteristics of IHS and Three Other Federal Health Care Programs (No. GAO-19-74R), 2018). By addressing the chronic underfunding of AI/AN health in the United States through the creation of the Federal Indian Health Insurance Plan, the IHS could allocate resources to address other challenges facing the organization, such as facilities upgrades and personnel recruitment. Looking toward the future, the IHS would continue to narrow its focus toward delivering high-quality, culturally congruent services in tribal lands, as well as in supporting public health initiatives.

We recognize as well that this solution is imperfect. It does not address the issue of health service delivery to members of federally-unrecognized tribes, currently left ineligible for IHS services. Population estimates for this community are not publicly available and are frequently shifting in size, but AI/AN members of unrecognized tribes likely number in the tens of thousands (Federal Funding for Non-Recognized Tribes (No. GAO-12-348), 2012). The future of the IHS itself will need to be addressed over time by tribal working groups and key stakeholders. The organization will need to address structural deficiencies highlighted in government reports, while building upon the cultural competence and resilience demonstrated in its efficient distribution of vaccines for COVID-19 (Hatzipanagos, 2021). AI/AN mistrust of the federal government, rooted in historical injustices, will also present a challenge to enrolling members to the new insurance program. We believe, however, that these challenges are not insurmountable. They can be met with productive engagement between the federal government and the Tribes, in creating a program that best serves AI/ANs today. In addition to working together to craft a culturally appropriate benefits package, such engagement may include ensuring AI/AN representation in the leadership personnel responsible for administering the program, as well as the establishment of a council composed of Tribal leaders offering ongoing consultation and direction to the program.

The Federal Indian Health Insurance Plan will thoroughly address the challenges faced by the IHS today. It took the *Patient Protection and Affordable Care Act of 2010* to assure the permanent reauthorization of the *Indian Health Care Improvement Act of 1976* and thereby of new programs and services within the IHS (Warne and Frizzell, 2014). It may well be that the Federal Indian Health Insurance Plan will only be realized during future health care reform. In the interim, however, sustained advocacy must be undertaken to keep the issue of AI/AN health prominent in the public consciousness. In doing so, our country may finally and honorably fulfill its solemn obligation to AI/AN communities.

## Declaration of Competing Interest

The authors declare the following financial interests/personal relationships which may be considered as potential competing interests: Dr. Khetpal reports working in an advisory role for Necessary Ventures. Mr. Roosevelt declares no conflicts of interest. Professor Adashi serves as a co-chair of the Safety Advisory Board of Ohana Biosciences, Inc.
